# Polymorphism of the dinuclear Co^III^–Schiff base complex [Co_2_(*o*-van-en)_3_]·4CH_3_CN (*o*-van-en is a salen-type ligand)

**DOI:** 10.1107/S2053229619003115

**Published:** 2019-03-20

**Authors:** Anna Vráblová, Juraj Černák, Larry R. Falvello, Milagros Tomás

**Affiliations:** a Department of Inorganic Chemistry, Institute of Chemistry, P. J. Šafárik University in Košice, Moyzesova 11, Košice, SK-04154, Slovakia; b Department of Inorganic Chemistry, University of Zaragoza, Pedro Cerbuna 12, Zaragoza, E-50009, Spain; c Aragón Materials Science Institute (ICMA), University of Zaragoza, Pedro Cerbuna 12, Zaragoza, E-50009, Spain; d Institute of Chemical Synthesis and Homogeneous Catalysis (ISQCH), University of Zaragoza, Pedro Cerbuna 12, Zaragoza, E-50009, Spain

**Keywords:** dinuclear cobalt(III), crystal structure, polymorphism, full interaction map, Schiff base ligand

## Abstract

Reactions of Co(OH)_2_ with the Schiff base bis­(2-hy­droxy-3-meth­oxy­benzyl­idene)ethyl­enedi­amine, denoted H_2_(*o*-van-en), under different conditions yielded the previously reported complex aqua­[bis­(3-meth­oxy-2-oxido­benzyl­idene)ethyl­enedi­amine]­cobalt(II), [Co(C_18_H_18_N_2_O_4_)(H_2_O)], **1**, under anaerobic conditions and two polymorphs of [μ-bis­(3-meth­oxy-2-oxido­benzyl­idene)ethyl­enedi­amine]­bis­{[bis­(3-meth­oxy-2-oxido­benzyl­idene)ethyl­enedi­amine]­cobalt(III)} aceto­nitrile tetra­solvate, [Co_2_(C_18_H_18_N_2_O_4_)_3_]·4CH_3_CN, *i.e.* monoclinic **2** and triclinic **3**, in the presence of air. Both novel polymorphs were chemically and spectroscopically characterized. Their crystal structures are built up of centrosymmetric dinuclear [Co_2_(*o*-van-en)_3_] complex mol­ecules, in which each Co^III^ atom is coordinated by one tetra­dentate dianionic *o*-van-en ligand in an uncommon bent fashion. The pseudo-­octa­hedral coordination of the Co^III^ atom is completed by one phenolate O and one amidic N atom of the same arm of the bridging *o*-van-en ligand. In addition, the asymmetric units of both polymorphs contain two aceto­nitrile solvent mol­ecules. The polymorphs differ in the packing orders of the dinuclear [Co_2_(*o*-van-en)_3_] complex mol­ecules, *i.e.* alternating *ABABAB* in **2** and *AAA* in **3**. In addition, differences in the conformations, the positions of the aceto­nitrile solvent mol­ecules and the pattern of inter­molecular inter­actions were observed. Hirshfeld surface analysis permits a qualitative inspection of the differences in the inter­molecular space in the two polymorphs. A knowledge-based study employing Full Inter­action Maps was used to elucidate possible reasons for the polymorphism.

## Abstract 2

Reactions of Co(OH)_2_ with the Schiff base bis­(2-hy­droxy-3-meth­oxy­benzyl­idene)ethyl­enedi­amine (*o*-van-en) yielded previously reported [Co^II^(*o*-van-en)(H_2_O)] under anaerobic conditions and two novel polymorphs of [Co^III^_2_(*o*-van-en)_3_]·4CH_3_CN in the presence of air. Structural data were used in a knowledge-based approach to elucidate the origin of the polymorphism.

## Introduction   

Polymorphism in the crystalline state – ‘the ability of a compound to crystallize in more than one crystal structure’ (Cruz-Cabeza *et al.*, 2015 ▸) – is important from a scientific, as well as from an industrial, point of view as sometimes subtle differences in the crystal structures of the polymorphs may lead to substanti­ally different properties. Such behaviour has been observed in the case of nonlinear optical materials (Munshi *et al.*, 2008 ▸), single mol­ecule magnets (Pavlov *et al.*, 2016 ▸), materials with spin-crossover (Tao *et al.*, 2012 ▸) or gas-absorption properties (Pal *et al.*, 2016 ▸), or the properties of pharmaceutically active materials (Covaci *et al.*, 2017 ▸; Rodríguez-Spong *et al.*, 2004 ▸; Potticary *et al.*, 2016 ▸), to mention a few examples. Recently, progress in the prediction of the crystal structures of polymorphs using solid-state density functional theory (DFT) simulations has been reported (Hasnip *et al.*, 2014 ▸).

Schiff bases in their deprotonated forms are widely used ligands as they may exhibit several potential coordination sites, which allows them to bond to one or more central metal atoms (Rezaeivala & Keypour, 2014 ▸; Vigato & Tamburini, 2004 ▸; Andruh, 2015 ▸). Reaction of ethane-1,2-di­amine with *o*-vanillin in a 1:2 molar ratio results in a Schiff base of the salen type, namely bis­(2-hy­droxy-3-meth­oxy­benzyl­idene)ethyl­ene­di­amine, denoted H_2_(*o*-van-en) (see Scheme 1). At present, more than 300 crystal structures, among them numerous complexes with transition metals and lanthanides (or their combinations), with this Schiff base are held in the Cambridge Structural Database (CSD; Groom *et al.*, 2016 ▸). Surprisingly, we find only one case for which polymorphism was reported, namely the copper complex [Cu(*o*-van-en)(H_2_O)]. One polymorph of this compound [CSD refcodes WICBIU (Saha *et al.*, 2007 ▸) and WICBIU01 (Odabaşoğlu *et al.*, 2007 ▸)] crystallizes in the ortho­rhom­bic space group *Pnma* with *Z*′ = 1, while the second crystallizes in the noncentrosymmetric ortho­rhom­bic space group *Pna*2_1_ with *Z*′ = 3 (refcode WICBIU02; Zhou *et al.*, 2015 ▸). It should be noted that the H_2_(*o*-van-en) Schiff base itself forms two known polymorphs whose formation can be considered as a consequence of two possible conformations of the ethane-1,2-di­amine part of the mol­ecule. Mol­ecules of H_2_(*o*-van-en) with an *anti* conformation of the ethane-1,2-di­amine fragment (VOJSUH; Cunningham *et al.*, 2004 ▸) crystallize in the monoclinic space group *P*2_1_/*n* (Cunningham *et al.*, 2004 ▸). In contrast, the polymorph crystallizing in the monoclinic space group *Pc* contains H_2_(*o*-van-en) mol­ecules with a *syn* arrangement of the ethane-1,2-di­amine fragment [refcodes VOJSUI (Mo *et al.*, 1990 ▸) and VOJSUI02 (Correia *et al.*, 2005 ▸)].
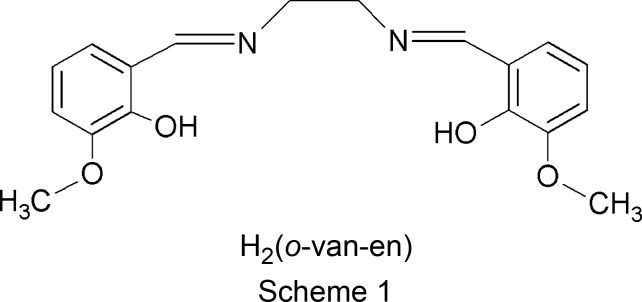


Within our broader study of Co^II^ complexes as magnetically active materials (Burzurí *et al.*, 2011 ▸; Smolko *et al.*, 2016 ▸), we have undertaken the study of the system formed by cobalt(II) hydroxide with the salen-type ligand *o*-van-en. From this system, depending on the experimental conditions, we have isolated three complexes, namely the previously reported [Co(*o*-van-en)(H_2_O)] (**1**) and two novel polymorphs of [Co_2_(*o*-van-en)_3_]·4CH_3_CN (**2** and **3**; see Scheme 2). The synthesis and crystal structure of **1** have already been reported (Jiang *et al.*, 2007 ▸). We report here a modified synthetic procedure leading to **1**, as well as the syntheses, crystal structures and comparisons of polymorphs **2** and **3**. We note that the analogous complex [Co_2_(*o*-van-en)_3_]·2Me_2_SO·2H_2_O with dimethyl sulfoxide and water solvent mol­ecules (whose content was not fully stoichiometric), was previously prepared and structurally characterized from photographic data (Calligaris *et al.*, 1970 ▸).

In seeking the factors responsible for polymorphism in [Co_2_(*o*-van-en)_3_]·4CH_3_CN, we undertook a study of the Full Inter­action Maps (FIMs) for the two structures (Wood *et al.*, 2013 ▸). These permitted what we believe is a plausible explanation for the origin of the polymorphism in this compound.
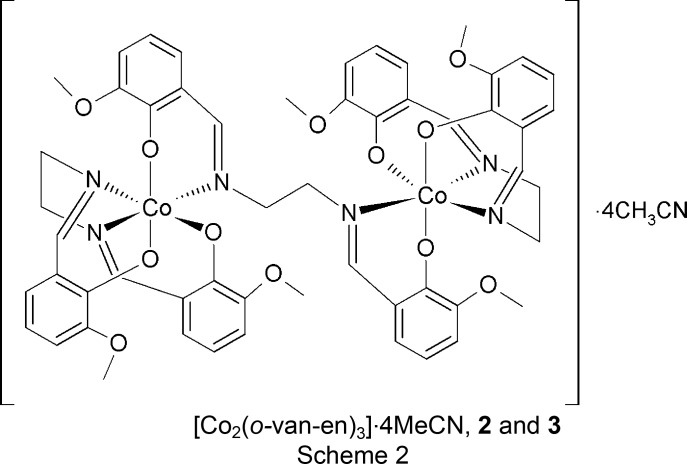


## Experimental   

### Materials   

H_2_(*o*-van-en) was synthesized using a slight modification of the procedure described by Ghose (1983 ▸, 1984 ▸), by the reaction of ethane-1,2-di­amine and *o*-vanillin in a 1:2 molar ratio under reflux conditions in ethanol. The remaining chemicals were used as received from commercial sources.

### Methods   

Elemental analyses (C, H and N) were performed on a PerkinElmer 2400 Series II CHNS/O analyser. IR spectra were recorded on a PerkinElmer Spectrum 100 CsI DTGS FT–IR spectrometer with a UATR 1 bounce-KRS-5 in the range 4000–300 cm^−1^ (UATR is a universal attenuated total reflectance accessory and KRS-5 is thallium bromo­iodide). The X-ray powder diffraction pattern of **1** was measured on a Rigaku D-Max/2500 diffractometer with a rotating anode and an RINT2000 vertical goniometer in the 2θ range 2.5–40° using Cu *K*α radiation (λ = 1.54178 Å) and a step size of 0.03°; the model powder diffraction pattern was calculated using the program *Mercury* (Macrae *et al.*, 2008 ▸).

For calculations of the Hirshfeld surfaces, the program *CrystalExplorer* was used (Spackman & Jayatilaka, 2009 ▸; Spackman & McKinnon, 2002 ▸). Full Inter­action Maps (FIMs) (Wood *et al.*, 2013 ▸) were calculated using the program *Mercury* (Macrae *et al.*, 2008 ▸).

### Synthesis and crystallization   

#### [Co(*o*-van-en)(H_2_O)], 1   

Solid Co(OH)_2_ (0.07 g, 0.76 mmol) was added to a de­oxy­genated water suspension of H_2_(*o*-van-en) (0.25 g, 0.76 mmol, 35 ml) under an inert argon atmosphere at room temperature. An orange solid appeared after a few minutes of stirring. The mixture was stirred overnight and the final dark-orange microcrystalline product **1** was filtered off, washed with water and dried in air (yield 80%, based on Co). Elemental analysis (%) calculated for C_18_H_20_CoN_2_O_5_: C 53.61, H 5.00, N 6.95; found: C 53.80, H 4.88, N 6.87. IR (ν/cm^−1^): 3315 (*b*), 3055 (*w*), 2899 (*w*), 2827 (*w*), 1651 (*m*), 1625 (*m*), 1600 (*m*), 1545 (*m*), 1468 (*m*), 1438 (*s*), 1391 (*m*), 1310 (*m*), 1239 (*s*), 1213 (*s*), 1169 (*m*), 1078 (*m*), 980 (*m*), 967 (*m*), 853 (*m*), 743 (*m*), 723 (*s*), 640 (*m*), 421 (*m*).

#### Monoclinic [Co_2_(*o*-van-en)_3_]·4CH_3_CN (Form I), 2   

The microcrystalline product **1** was dissolved in aceto­nitrile in air with stirring at room temperature and after dissolution was left aside for crystallization. Within a few hours, the resulting solution had changed colour from dark red to brown–black. Black block-shaped crystals of **2** were obtained after a few days. As the crystals were unstable when separated from the mother liquor, presumably due to loss of solvent mol­ecules, they were mounted for diffraction data collection immediately after removal from the mother liquor.

#### Triclinic [Co_2_(*o*-van-en)_3_]·4CH_3_CN (Form II), 3   

Solid Co(OH)_2_ (0.07 g, 0.76 mmol) was added to a water suspension of H_2_(*o*-van-en) (0.25 g, 0.76 mmol, 20 ml) at room temperature in air and stirred overnight until the microcrystalline solid had changed colour from yellow–brown to black. The product thus formed was filtered off, dried in air and recrystallized from hot aceto­nitrile solution (∼60–70 °C). Black block-shaped crystals of **3** appeared after a few days. The crystals of **3** were not stable in air, so they were mounted for diffraction data collection immediately after removal from the aceto­nitrile solution.

### Refinement   

Crystal data and global indicators from the structure refinements are collected in Table 1 ▸. For polymorph **2**, nonmethyl H atoms were located in a difference map and refined freely with individual variable isotropic displacement parameters. Methyl H atoms were initially placed at positions derived from difference electron-density maps, with C—H = 0.98 Å, and were refined as riders, with *U*_iso_(H) = 1.5*U*_eq_ of their respective bonding partners; the methyl groups were allowed to rotate but not tilt. The disordered methyl group at C1 was split into two parts, both with half occupancy.

In polymorph **3**, the H atoms bonded to imine atoms C8, C11 and C26 were found in a difference map and refined freely. The remaining nonmethyl H atoms, as well as the methyl H atoms of the complex (at C1, C18 and C19), were placed at calculated positions (C—H = 0.99, 0.98 and 0.95 Å for methyl­ene, methyl and aromatic H atoms, respectively). The methyl H atoms of the CH_3_CN mol­ecules were placed at positions derived from a difference map and refined as riders which were permitted to rotate but not tilt. The disordered MeCN mol­ecule was split into two parts with occupancies constrained to sum to unity and with C—C and C—N bonds restrained to be the same lengths in both congeners. For the H atoms, *U*_iso_ values were set at *xU*_eq_ of their respective bonding partners, with *x* = 1.2 for nonmethyl H atoms and the methyl group at C28, and *x* = 1.5 for the remaining methyl groups.

## Results and discussion   

### Syntheses and identification   

Jiang *et al.* (2007 ▸) reported the crystal structure and the *in situ* solvothermal synthesis of the complex [Co(*o*-van-en)(H_2_O)], **1**, starting from 2-hy­droxy-3-meth­oxy­benzaldehyde, ethane-1,2-di­amine and cobalt(II) nitrate. We have prepared the same product in microcrystalline form by direct reaction of cobalt(II) hydroxide with the Schiff base H_2_(*o*-van-en) under mild conditions and an inert argon atmosphere. Le Bail refinement (Fig. S1 in the supporting information) of the measured X-ray diffraction pattern of **1** using the program *JANA2006* (Le Bail *et al.*, 1988 ▸; Petříček *et al.*, 2014 ▸) corroborated the phase purity and the identity of our product **1** with that reported by Jiang *et al.* (2007 ▸). In addition, the identity and the phase purity of **1** were further confirmed by the results of the elemental analysis.

An attempt to recrystallize microcrystalline product **1** from aceto­nitrile at room temperature in the presence of air led to oxidation of Co^II^ to Co^III^ and the formation of the monoclinic form (Form I, **2**) of [Co_2_(*o*-van-en)_3_]·4CH_3_CN. Direct reaction of the Schiff base with Co(OH)_2_ in the presence of air led to a black microcrystalline crude product, clearly indicating oxidation of the starting Co^II^ to Co^III^. When the resulting crude product was recrystallized from hot aceto­nitrile, crystals of the triclinic form (Form II, **3**) of [Co_2_(*o*-van-en)_3_]·4CH_3_CN separated out. We note that Calligaris *et al.* (1970 ▸) prepared single crystals of the analogous complex [Co_2_(*o*-van-en)_3_]·2Me_2_SO·2H_2_O with dimethyl sulfoxide and water solvent mol­ecules starting from the Co^II^ complex **1**.

### Crystal structures   

The mol­ecular and crystal structure of **1** was reported by Jiang *et al.* (2007 ▸). The central Co^II^ atom in **1** is penta­coordinate, with the donor atoms from the Schiff base occupying the basal plane of the square pyramid, while the apical position is occupied by an aqua ligand (Fig. S2 in the supporting information).

**Figure 1:**
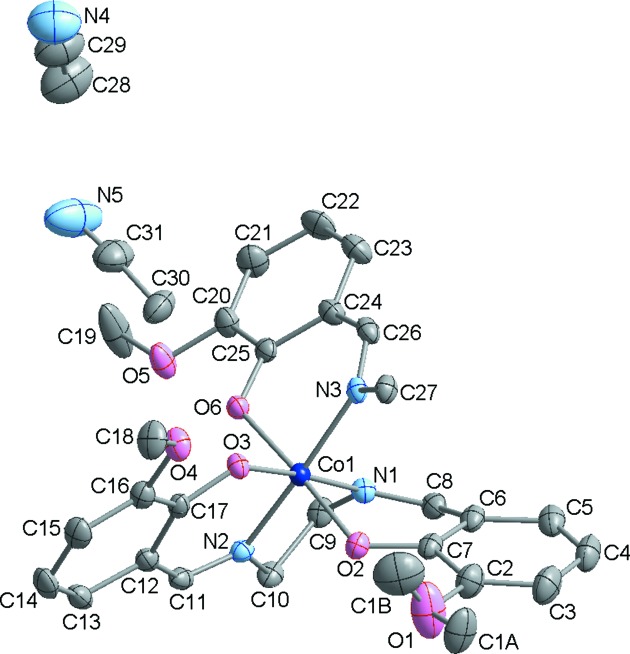
The asymmetric unit of **2**, with the atom-numbering scheme. Displacement ellipsoids are drawn at the 50% probability level. Half of the dinuclear mol­ecule (without H atoms) is depicted for the sake of clarity.

Form I of [Co_2_(*o*-van-en)_3_]·4CH_3_CN (polymorph **2**) crystallizes in the monoclinic space group *P*2_1_/*c*, while Form II (polymorph **3**) crystallizes in the triclinic space group *P* 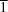. The crystal structures of both **2** and **3** are built up of centrosymmetric dinuclear [Co_2_(*o*-van-en)_3_] complex mol­ecules; the triclinic form contains one dinuclear centrosymmetric mol­ecule in the unit cell (*Z* = 1), while for the monoclinic form, *Z* = 2 (Figs. 1 ▸ and 2 ▸, respectively). In both polymorphs, the Co^III^ atoms are coordinated by one tetra­dentate *o*-van-en ligand in an uncommon bent fashion. A similar bent coordination was reported for [Fe_2_(*o*-van-en)_3_]·CH_2_Cl_2_·0.5H_2_O (Costes *et al.*, 2010 ▸). The pseudo-­octa­hedral coordination environments of the Co^III^ atoms are completed by one phenolate O and one imine N atom placed in *cis* positons and originating from the same arm of the bridging *o*-van-en ligand. In addition, the asymmetric units of both polymorphs contain two aceto­nitrile (MeCN) solvent mol­ecules. The crystal structure of [Co_2_(*o*-van-en)_3_]·2Me_2_SO·2H_2_O (CSD refcode COMSAL; Calligaris *et al.*, 1970 ▸) contains the same complex mol­ecule; the atomic coordinates are not available for COMSAL, obviating a closer comparison with our two polymorphs.

The Co—O and Co—N bond lengths in **2** and **3** (see Table 2 ▸) are in line with those found for similar Co^III^ complexes, *e.g.* in [Co(salen)(acac)]·1.5H_2_O (acac is acetyl acetate; Bailey *et al.*, 1972 ▸) and [Co(salen)(acac)]·0.7H_2_O (Calligaris *et al.*, 1972 ▸). The values found are, as expected, somewhat shorter than those reported for Co^II^ complex **1**, in line with the smaller ionic radius of the Co^III^ atom (Shannon, 1976 ▸).

**Figure 3:**
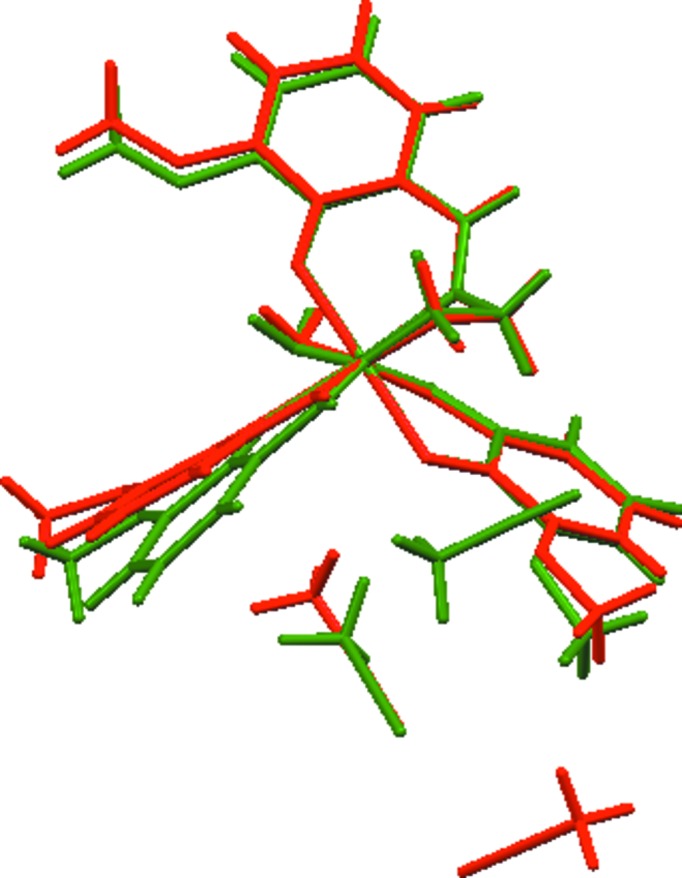
Wire models of the mol­ecular structures of polymorphs **2** (red) and **3** (green). The structures are superimposed in such a way that the positions of the central Co atoms, as well as the donor atoms, are overlapped as nearly as possible. Only the asymmetric part of the dinuclear complex mol­ecule is shown for clarity.

The dinuclear complex mol­ecules in the two polymorphs display small but significant differences with respect to their geometrical parameters, and these can be clearly seen in Fig. 3 ▸. For example, the torsion angle O3—Co1—N2—C11 in Form I exhibits a value of 2.1 (2)°, in contrast to the corresponding value of 12.06 (19)° in Form II; as a consequence, the C12–C17 aromatic rings form different angles with the Co1/O3/N3/N1/N2 equatorial plane in the respective polymorphs, *i.e.* 6.89° in **2** *versus* 15.70° in **3** (Fig. S3 in the supporting information). As for the meth­oxy groups within the ligand, the most striking difference between Forms I and II is that in Form I, the O1 meth­oxy group is positionally disordered two ways, with occupancies set to half (Fig. 1 ▸). As for the remaining two meth­oxy groups, *i.e.* involving atoms O4 and O5, those with O5 display a small conformational difference in the two polymorphs, as can be seen by a comparison of the respective C19—O5—C20—C21 torsion angles, exhibiting values of 7.2 (4) (Form I) and 13.44 (1)° (Form II).

**Figure 4:**
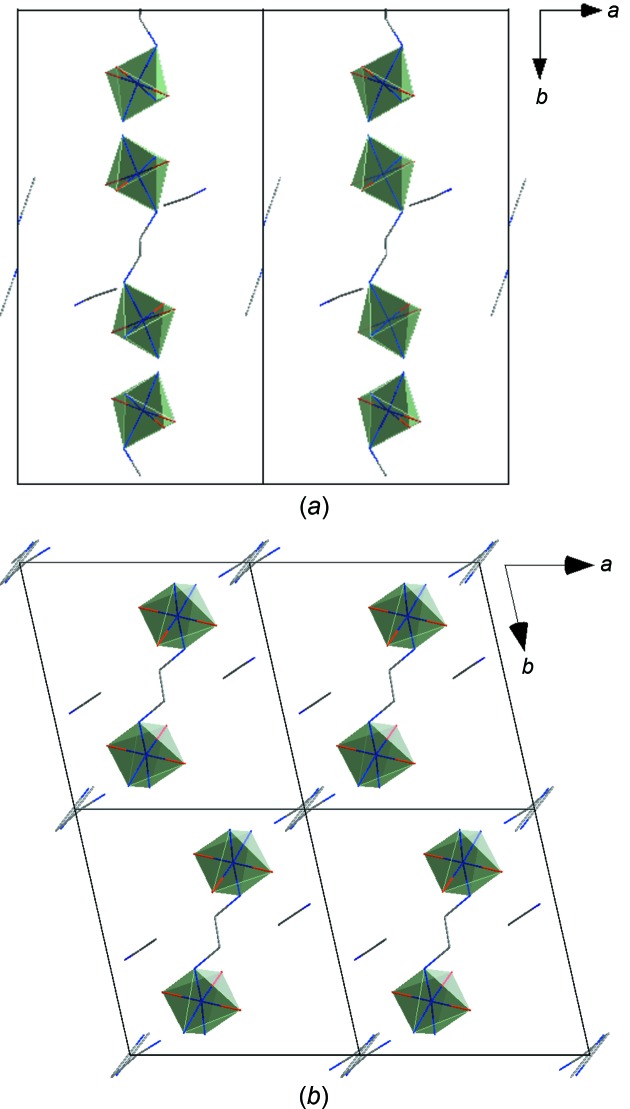
Packing in the crystal structures of (*a*) **2** and (*b*) **3**, each viewed along its *c* axis. For clarity, only coordination polyhedra, bridges linking the polyhedra and aceto­nitrile solvent mol­ecules are shown.

Packing diagrams for polymorphs **2** and **3** are shown in Fig. 4 ▸, in which the dinuclear [Co_2_(*o*-van-en)_3_] complex mol­ecules are represented by CoO_3_N_3_ octa­hedra connected by four-atom centrosymmetric N3—C27—C27^i^—N3^i^ bridges. The higher symmetry of Form I (monoclinic) coincides with a doubling of its unit-cell volume with respect to that of triclinic Form II. Moreover, the 2_1_ screw axis parallel to *b* in Form I generates an alternating *ABABAB* stacking pattern, in contrast to Form II, in which the dinuclear units are arranged in a simple *AAA* manner. We note also that the MeCN solvent mol­ecules occupy slightly different positions relative to the main mol­ecules in the two polymorphs.

In what follows, we find it convenient to distinguish among hydrogen bonds of differing strengths and to treat these as distinct from contacts that may be adventitious and of questionable structure-directing capacity. For the purposes of this discussion, hydrogen bonds in which O and/or N atoms are both donors and acceptors will be called classical hydrogen bonds, those with imino or aromatic C—H groups as donors will be called nonclassical hydrogen bonds or weak hydrogen bonds and contacts involving methyl or methyl­ene in donor roles will be called simply contacts, with no attempt at a more nuanced discrimination between what can and cannot be called a hydrogen bond. We will use a nonrigorous criterion, namely the default limits used by the program *PLATON* (Spek, 2009 ▸), to draw a convenient line between what we do and do not denote as hydrogen bonds – again, for the purposes of this discussion.

In both polymorphs, there are no classical hydrogen bonds due to the lack of suitable donors after deprotonation of the hydroxide groups. Surprisingly, among the weak hydrogen-bonding inter­actions of the =C—H⋯*A* or C_ar_—H⋯*A* types (*A* = O or N), there is only one such hydrogen bond in each of the polymorphs, in both cases inter­molecular.

**Figure 5:**
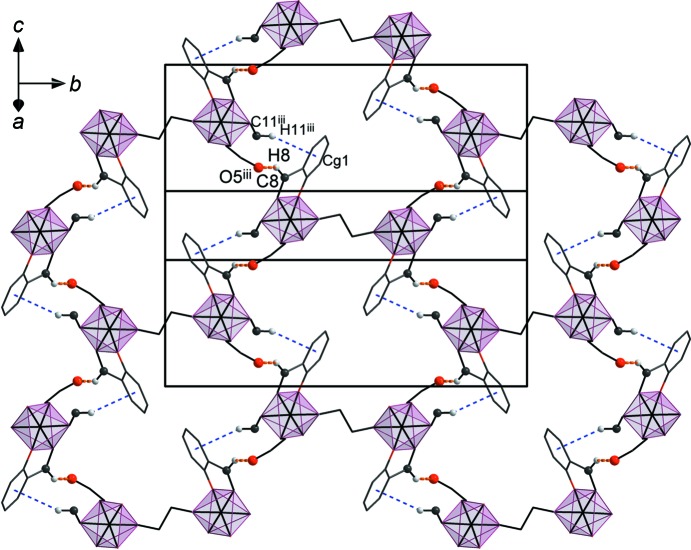
The supra­molecular layer formed by weak inter­molecular C—H⋯O hydrogen bonds (C8—H8⋯O5^iii^; orange dashed lines) and C—H⋯π inter­actions (C11^iii^—H11^iii^⋯*Cg*1; blue dashed line) in **2**. The view is in the *bc* plane. *Cg*1 represents the centre of gravity of the C2–C7 aromatic ring. [Symmetry code: (iii) *x*, −*y* + 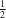, *z* + 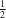.]

In polymorph **2** (Form I), the only weak hydrogen-bonding inter­action is C8—H8⋯O5^iii^ with participation of the imine H atom (see Table 3 ▸ for symmetry code). This inter­action links the complex mol­ecules into supra­molecular layers in the *bc* plane (Fig. 5 ▸). The C11—H11 bond of the other imine group is directed toward the π-system of the C2^iii^–C7^iii^ aromatic ring; this additional weak C—H⋯π inter­action has an H⋯*Cg* distance of 2.91 (3) Å and a γ angle between the *Cg*—H vector and ring normal of 12.41°. As can be seen from Fig. 5 ▸, this inter­action serves to reinforce the hydrogen-bonding inter­action mentioned above. We note that additional inter­molecular contacts of the C—H⋯*X* (*X* = N and O) type, with H atoms from the methoxy methyl group (C1*B*) or the MeCN solvent mol­ecules (C28 and C30) can also be considered to contribute to the inter­molecular cohesion (Table 3 ▸) and likewise for close contacts of the C—H⋯π type with participation of (C10—)H10 methyl­ene and (C28—)H28*A* methyl H atoms (Table S1 in the supporting information). In Form I, there is no classical intra­molecular hydrogen bonding, unless one considers close contacts of the C—H⋯O type with H atoms from a meth­oxy group (disordered C1 atom in position *B*) or methyl­ene groups (C9 and C27; Table 3 ▸ and Fig. S4 in the supporting information) to be significant; these may help to stabilize the conformation of the complex mol­ecule.

**Figure 6:**
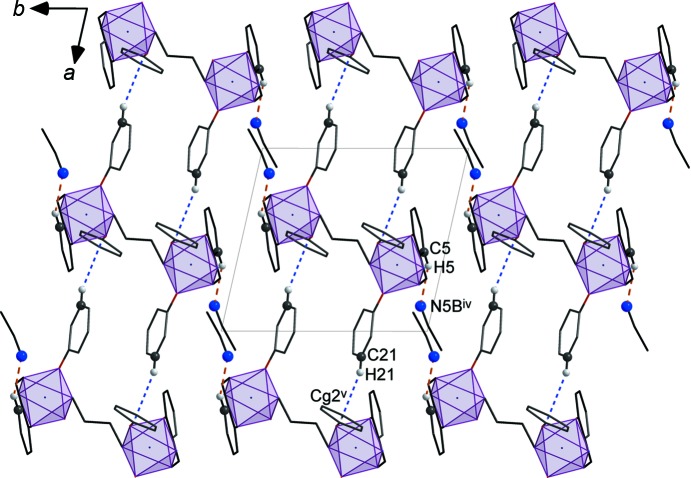
Packing mediated by the C5—H5⋯N5*B* ^iv^ hydrogen bond (orange dashed line) and the C21_ar_—H21⋯*Cg*2^v^ inter­action (blue dashed line) in **3**. *Cg*2 represents the centre of gravity of the C12–C17 aromatic ring. [Symmetry codes: (iv) −*x* + 2, −*y*, −*z* + 1; (v) *x* + 1, *y*, *z*.]

Similarly, in Form II, only one nonclassical hydrogen bond of the C_ar_—H⋯N type, namely C5—H5⋯N5*B* ^iv^ is present (Table 4 ▸ and Fig. 6 ▸) and this links the complex mol­ecules with the disordered MeCN solvent mol­ecule in the more populated position (N5*B*). As in Form I, there is one weak C—H⋯π inter­action in which are involved the H21 atom from the aromatic ring as donor and the π-system of the C12^v^–C17^v^ aromatic ring as acceptor; the H⋯*Cg* distance is 2.64 Å and the γ angle between the *Cg*—H vector and the ring normal is 8.82°. This inter­action links the complex mol­ecules into supra­molecular chains running along the *a* axis (Fig. 6 ▸). The crystal packing is also stabilized by additional contacts of the C—H⋯*X* (*X* = O and N) and C—H⋯π types coming from both MeCN solvent mol­ecules and involving their methyl groups (Table 4 ▸) and the C9—H9*A* methyl­ene group near the C20^iv^–C25^iv^ aromatic ring (Table S2 in the supporting information). As for the intra­molecular inter­actions, these include only contacts of the C—H⋯O type involving H atoms from methyl­ene groups of the ligand (Table 4 ▸ and Fig. S5 in the supporting information).

With the aim of elucidating the factors responsible for the polymorphism of this system, we examined the packing patterns of structures **2** and **3** further using Hirshfeld surfaces (Spackman & Jayatilaka, 2009 ▸) and Full Inter­action Maps (FIMs; Wood *et al.*, 2013 ▸).

**Figure 7:**
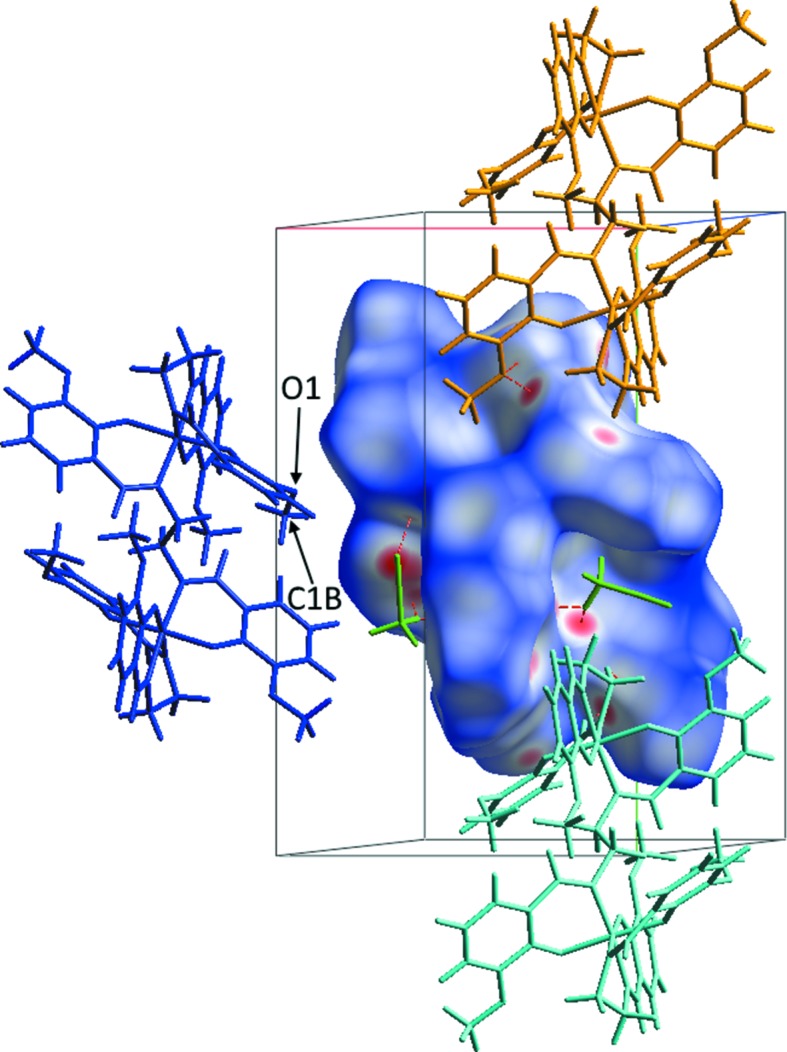
Hirshfeld surface of the dinuclear complex mol­ecule of **2** plotted over *d*_norm_ (normalized contact distance) from −0.4000 to 1.5000 a.u., indicating the complex mol­ecule with the disordered C1*B* methyl group in position *B*. The MeCN solvent mol­ecules are displayed in green. Close contacts (O⋯H ≤ 2.60 Å and N⋯H ≤ 2.63 Å) are shown as red dashed lines. Neighbouring mol­ecules are drawn using wire models in different colours in order to give a better view of the contacts.

The Hirshfeld surfaces for polymorphs **2** and **3** (Figs. 7 ▸ and 8 ▸, respectively) provide a concise visual indication that the inter­molecular inter­actions are different in the two structures. In both cases, the structures suffer minor disorder, which complicates the preparation and inter­pretation of the Hirshfeld surfaces and fingerprint plots. This arises because the simultaneous presence of two disorder groups of the same disorder assembly will generate the appearance of artificial and impossibly short contacts in the Hirshfeld surfaces and fingerprint plots. At the same time, these tools do permit further discussion of the hydrogen bonds and other contacts. A full description and inter­pretation of the plots is given in the supporting information. We provide here only the aspects relevant to the present discussion.

**Figure 8:**
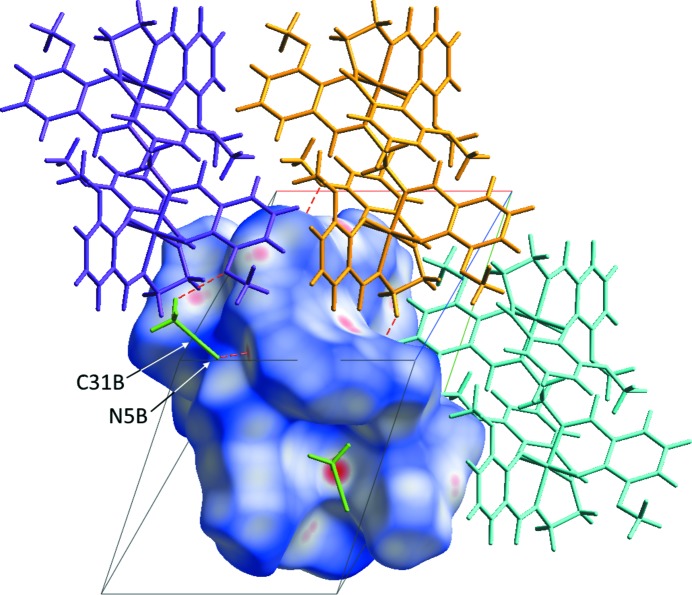
Hirshfeld surface diagram of the dinuclear complex mol­ecule of **3** plotted over *d*_norm_ (normalized contact distance) from −0.4000 to 1.5000 a.u., indicating the disordered MeCN mol­ecule in position *B*. The MeCN solvent mol­ecules are displayed in green. Neighbouring complex mol­ecules are shown in different colours using a wire model. Close contacts are displayed as red dashed lines, *i.e.* O⋯H ≤ 2.60 Å and N⋯H ≤ 2.63 Å.

Fig. 7 ▸ shows the Hirshfeld surface for one of the disordered congeners from structure **2** (for the second disordered congener, see Fig. S6 in the supporting information), for which no impossibly short contacts are generated. Fig. 8 ▸ shows an analogous plot for structure **3** (Fig. S7 gives the analogous plot for the second disordered congener of **3**). The distributions of favourable structure-stabilizing contacts (red areas) in the two plots give a clear qualitative indication that the inter­molecular spaces in the two structures are organized in different fashions. This does not give us a clear indication of the origins of the polymorphism. To explore that question, we undertook an examination of FIMs.

The FIM (Wood *et al.*, 2013 ▸) is a knowledge-based tool that provides a visual map of the frequencies with which the chemical fragments or functional groups in a given structure have been observed to inter­act with different types of neighbours – for example, with hydrogen-bond donors or acceptors. The map is assembled using the crystal structure information held in the Cambridge Structural Database (Groom *et al.*, 2016 ▸), which as of this writing is approaching one million structures. We constructed the FIMs for structures **2** and **3** (Forms I and II, respectively) to see if indeed more than one possible favourable donor–acceptor set could be identified. This would indicate that more than one arrangement of mol­ecules in a crystal would produce stabilizing inter­actions.

The deprotonated H_2_(*o*-van-en) mol­ecule contains two potential donor sites at imine C atoms (Scheme 3, red arrows) and six acceptor sites (Scheme 3, blue arrows). Only two of the acceptor sites, namely the meth­oxy O atoms, are good candidates for inter­molecular inter­actions, since the N atoms of the imine groups and the O atoms of the deprotonated hy­droxy groups are occupied in coordination to the central Co^III^ atom.

**Figure 9:**
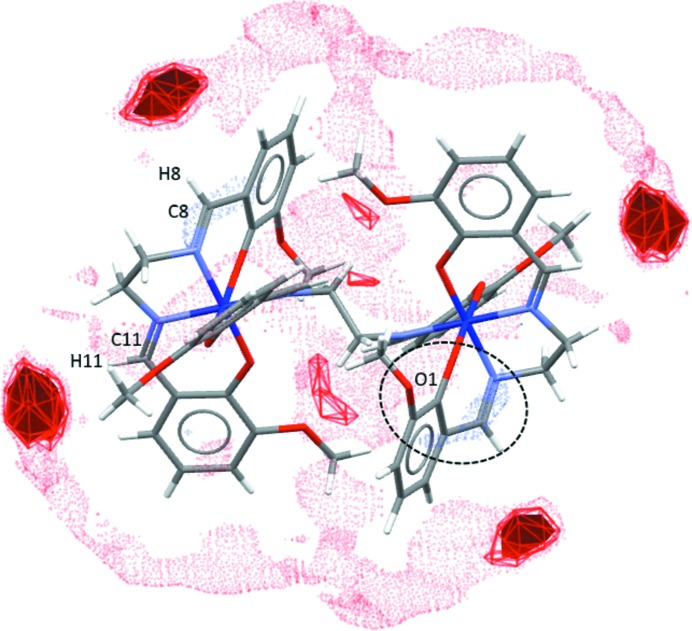
The FIM for complex mol­ecules of **2**. Hydrogen-bond-donor regions are represented in blue and acceptor regions are represented in red. Dots, wireframe and solid regions represent frequencies of ×2, ×4 and ×6 those expected for a random distribution of contacts.

For the FIM of polymorph **2**, we used only one disordered position of the meth­oxy group (atoms C1*B*, H1*BA*, H1*BB* and H1*BC*, with site-occupancy factors of 50%). The FIM displays four strong red (hydrogen-bond acceptor) regions, two of them symmetry independent (Fig. 9 ▸). They represent hydro­gen-bond acceptors in the vicinity of the imine C—H group of the distal ligand. Furthermore, we observe a weak blue region near the potential acceptor site at meth­oxy atom O1 (circled in Fig. 9 ▸). The O atom of this orientation of the meth­oxy group is more exposed on the surface of the complex mol­ecule, which is also reflected on the corresponding FIM (Fig. 9 ▸).
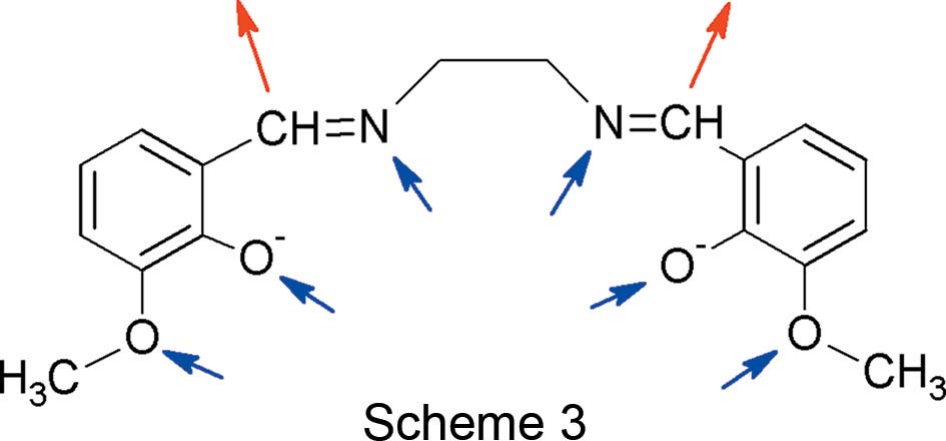


**Figure 10:**
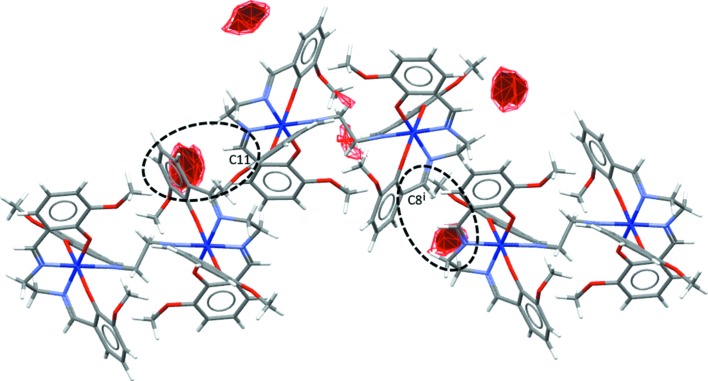
The environments of the two symmetrically independent donor regions in the FIM of polymorph **2**. Only the ×4 and ×6 levels are shown.

In polymorph **2** (Form I), the acceptor region near the donor C11—H11 imine group, pictured in the FIM as a red region, is occupied by the electron density of the π-system of the C2^iii^–C7^iii^ aromatic ring, and this contact, as described above, can be considered to be a weak C—H⋯π inter­action [H⋯*Cg* = 2.91 (3) Å and γ angle between *Cg*—H vector and ring normal of 12.41°; left circle in Fig. 10 ▸]. The acceptor region for the C8—H8 imine group is not occupied by any acceptor group (right circle in Fig. 10 ▸).

**Figure 11:**
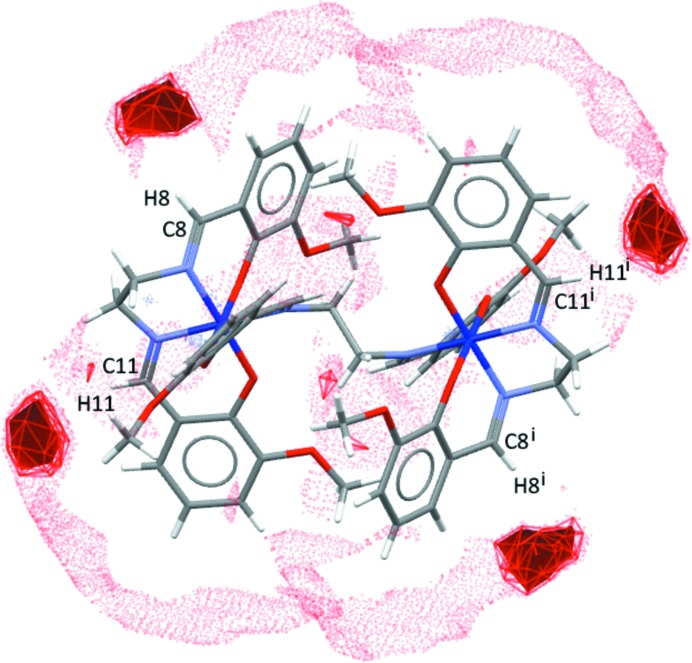
The FIM for complex mol­ecules of polymorph **3**. Hydrogen-bond-donor regions are represented in blue and acceptor regions are represented in red. Dots, wireframe and solid regions represent frequencies of ×2, ×4 and ×6 those expected for a random distribution of contacts.

As seen for polymorph **2** (Form I), the FIM of the main mol­ecule in polymorph **3** (Form II) shows four regions where the presence of hydrogen-bond acceptors is favoured, as observed in previously determined crystal structures (red regions in Fig. 11 ▸, two per asymmetric unit), reflecting the donor capabilities of the imine C8—H8 and C11—H11 groups and their symmetry relatives. The only possible acceptor sites (meth­oxy and oxy groups) are oriented toward the inter­ior of the complex mol­ecule, mostly forming intra­molecular inter­actions, so that the FIM does not show any potential donor region.

**Figure 12:**
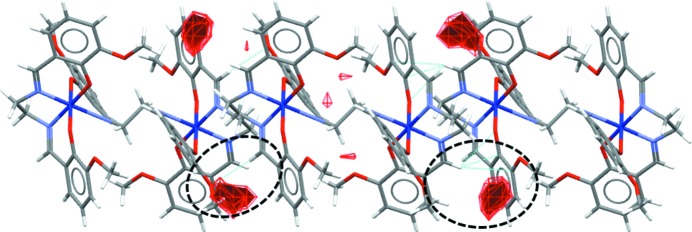
The occupation of two symmetrically independent donor regions in the FIM of polymorph **3**. Only ×4 and ×6 levels are shown.

In polymorph **3**, the donor C8—H8 imine group is involved in a rather weak inter­molecular close contact (C8—H8⋯O5^iv^; left circled inter­action in Fig. 12 ▸), beyond the default limits of *PLATON* (Spek, 2009 ▸), and similarly, the donor site at the C11—H11 imine group is involved in a weak C—H⋯π inter­action (right circled inter­action in Fig. 12 ▸), also beyond the conventional limits of most programs. It is suggested that these inter­actions, weak though they be, act as directors for the packing of complex mol­ecules in the structure of **3**.

In general, in **2** and **3**, the strongest regions of inter­molecular inter­actions in the FIMs are occupied by symmetry-related mol­ecules mediated by C—H⋯O-type hydrogen bonds and C—H⋯π inter­actions – or not occupied at all. None of the inter­actions mentioned lies exactly in the inter­action region (the acceptor is too far away). MeCN mol­ecules do not enter the donor or acceptor regions of the complex mol­ecules in either of these two polymorphs.

The FIMs suggest that this mol­ecule does not possess a strong capacity for self-recognition with significant inter­actions. In the configurations found in Forms I and II, four regions with a significant capacity for hydrogen-bond donation are not matched by any segments with the corresponding capacity to accept hydrogen bonds. So the polymorphism is not a result of a surfeit of mol­ecular arrangements leading to highly stabilizing inter­actions. Rather, we conclude that the cohesion in the crystals of **2** and **3** is a result of energetically poorer inter­actions. And it is not surprising that there would be more than one way to achieve a lesser level of stability. We note again here that crystals of both polymorphs are unstable outside of their mother liquids at the temperature at which they are formed.

## Conclusion   

From the system Co(OH)_2_ + H_2_(*o*-van-en) under different experimental conditions, two cobalt complexes were isolated in a total of three solid forms – under anaerobic conditions, the already structurally characterized Co^II^ complex **1**, and in the presence of air, two novel Co^III^-containing solids **2** and **3**. The new complexes were chemically and spectroscopically characterized. Products **2** and **3** are monoclinic and triclinic polymorphs, respectively, and both are formed by centrosymmetric dinuclear [Co_2_(*o*-van-en)_3_] complex mol­ecules in which two tetra­dentate *o*-van-en ligands chelate the two hexa­coordinated Co^III^ atoms, while the remaining *o*-van-en ligand bridges the two Co^III^ atoms in a bis-chelate fashion. The com­position of both polymorphs is completed by two MeCN solvent mol­ecules. The two polymorphs differ in the packing of the dinuclear [Co_2_(*o*-van-en)_3_] complex mol­ecules and con­formational differences in the complex mol­ecules were also observed. The Hirshfeld surfaces reflect the observed disorder for both polymorphs and suggest possible reasons for it; they also confirm the presence of contacts represented by weak hydrogen-bonding inter­actions, and they further indicate that the MeCN mol­ecules play a role in the packing as they fill the hollows formed between the packed complex mol­ecules. The FIMs of both polymorphs show that the regions of inter­molecular inter­actions are occupied by congeners of the complex, leaving unrequited hydrogen-bonding capability and suggesting an explanation for the polymorphism. Furthermore, aceto­nitrile solvent mol­ecules as rich electron donors do not enter the acceptor regions of the complex in either of the two polymorphs. These observations corroborate the observed low stability of both polymorphs with respect to the loss of their solvent mol­ecules.

## Supplementary Material

Crystal structure: contains datablock(s) 2, 3, global. DOI: 10.1107/S2053229619003115/ky3166sup1.cif

Structure factors: contains datablock(s) 2. DOI: 10.1107/S2053229619003115/ky31662sup2.hkl

Structure factors: contains datablock(s) 3. DOI: 10.1107/S2053229619003115/ky31663sup3.hkl

Additional figures and details of the Hirshfeld surface analysis. DOI: 10.1107/S2053229619003115/ky3166sup4.pdf

CCDC references: 1900885, 1900884

## Figures and Tables

**Figure 2:**
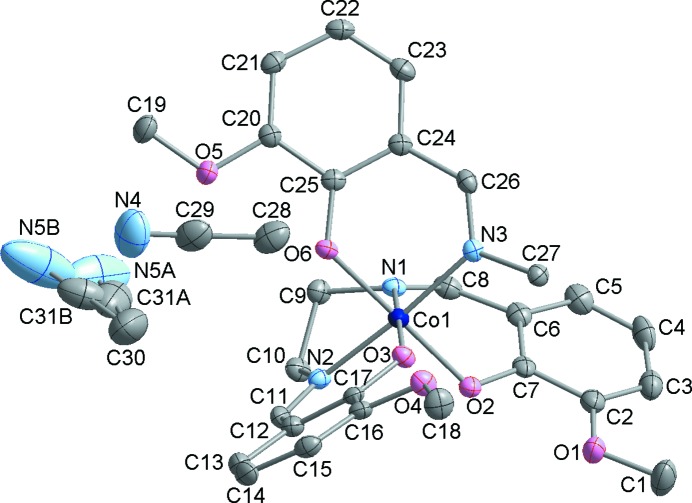
The asymmetric unit of **3**, with the atom-numbering scheme. Displacement ellipsoids are drawn at the 50% probability level. Only the half of the dinuclear mol­ecule present in the asymmetric unit is drawn (without H atoms) for the sake of clarity.

**Table 1:** Experimental details

	**2** (Form I)	**3** (Form II)
Crystal data		
Chemical formula	[Co_2_(C_18_H_18_N_2_O_4_)_3_]·4C_2_H_3_N	[Co_2_(C_18_H_18_N_2_O_4_)_3_]·4C_2_H_3_N
*M*_r_	1261.10	1261.10
Crystal system, space group	Monoclinic, *P*2_1_/*c*	Triclinic, *P* 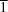
Temperature (K)	100	100
*a*, *b*, *c* (Å)	12.4355 (3), 21.6501 (4), 11.9462 (3)	10.4971 (5), 11.6195 (5), 13.7158 (6)
α, β, γ (°)	90, 115.364 (4), 90	70.471 (4), 71.667 (4), 72.466 (4)
*V* (Å^3^)	2906.25 (15)	1460.26 (13)
*Z*	2	1
Radiation type	Mo *K*α	Mo *K*α
μ (mm^−1^)	0.64	0.64
Crystal size (mm)	0.19 × 0.17 × 0.04	0.21 × 0.14 × 0.05

Data collection		
Diffractometer	Rigaku Xcalibur Sapphire3	Rigaku Xcalibur Sapphire3
Absorption correction	Multi-scan (*CrysAlis PRO*; Rigaku OD, 2015 ▸)	Multi-scan (*CrysAlis PRO*; Rigaku OD, 2015 ▸)
*T*_min_, *T*_max_	0.925, 1.000	0.835, 1.000
No. of measured, independent and observed [*I* > 2σ(*I*)] reflections	24772, 6530, 4887	20710, 6041, 5192
*R*_int_	0.061	0.039
(sin θ/λ)_max_ (Å^−1^)	0.650	0.628

Refinement		
*R*[*F* ^2^ > 2σ(*F* ^2^)], *wR*(*F* ^2^), *S*	0.045, 0.106, 1.02	0.037, 0.094, 1.04
No. of reflections	6530	6041
No. of parameters	475	421
No. of restraints	0	3
H-atom treatment	H atoms treated by a mixture of independent and constrained refinement	H atoms treated by a mixture of independent and constrained refinement
Δρ_max_, Δρ_min_ (e Å^−3^)	0.54, −0.39	0.89, −0.35

Computer programs: *CrysAlis PRO* (Rigaku OD, 2015 ▸), *SHELXT2014* (Sheldrick, 2015*a* ▸), *DIAMOND* (Brandenburg & Putz, 1999 ▸), *Mercury* (Macrae *et al.*, 2008 ▸), *SHELXL2018* (Sheldrick, 2015*b* ▸), *WinGX* (Farrugia, 2012 ▸), *PARST* (Nardelli, 1983 ▸) and *CrystalExplorer* (Spackman & Jayatilaka, 2009 ▸).

**Table 2:** Selected bond lengths (Å) for **2** and **3**

	**2**	**3**
Co1—O3	1.8915 (16)	1.8966 (13)
Co1—O2	1.9073 (17)	1.9002 (13)
Co1—O6	1.9093 (16)	1.9118 (13)
Co1—N1	1.8894 (19)	1.8993 (16)
Co1—N2	1.9041 (19)	1.9133 (16)
Co1—N3	1.9445 (19)	1.9271 (16)

**Table 3:** Hydrogen-bond geometry (Å, °) for **2** [Chem scheme1]

*D*—H⋯*A*	*D*—H	H⋯*A*	*D*⋯*A*	*D*—H⋯*A*
C1*B*—H1*BA*⋯N4^ii^	0.98	2.59	3.333 (10)	133
C1*B*—H1*BB*⋯O2	0.98	2.44	2.985 (8)	115
C8—H8⋯O5^iii^	0.96 (3)	2.34 (3)	3.119 (3)	138 (2)
C9—H9*B*⋯O6	1.00 (3)	2.51 (2)	2.924 (3)	104.6 (17)
C27—H27*A*⋯O2	0.94 (2)	2.45 (2)	3.042 (3)	121.1 (18)
C27—H27*B*⋯O3^i^	0.93 (3)	2.53 (2)	3.180 (3)	127.5 (18)
C28^ii^—H28*C* ^ii^⋯O1	0.98	2.52	3.266 (5)	133
C30—H30*C*⋯O3	0.98	2.52	3.188 (3)	126
C30—H30*C*⋯O6	0.98	2.34	3.245 (3)	153

Symmetry codes: (i) 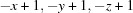; (ii) 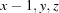; (iii) 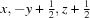.

**Table 4:** Hydrogen-bond geometry (Å, °) for **3** [Chem scheme1]

*D*—H⋯*A*	*D*—H	H⋯*A*	*D*⋯*A*	*D*—H⋯*A*
C5—H5⋯N5*B* ^iv^	0.95	2.57	3.454 (19)	156
C9—H9*B*⋯O6	0.99	2.50	2.955 (2)	107
C27—H27*A*⋯O3^i^	0.99	2.50	3.148 (2)	123
C27—H27*A*⋯O4^i^	0.99	2.56	3.463 (2)	151
C27—H27*B*⋯O2	0.99	2.40	2.980 (2)	117
C28—H28*A*⋯O6	0.98	2.29	3.256 (3)	169
C30—H30*D*⋯N4	0.98	2.56	3.451 (5)	151

Symmetry codes: (i) 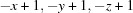; (iv) 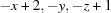.
