# Supplementary material for: Polymorphism of the dinuclear CoIII–Schiff base complex [Co2(o-van-en)3]·4CH3CN (o-van-en is a salen-type ligand)
Source: Acta Crystallogr C Struct Chem. 2019 Mar 20;75(Pt 4):433–42. doi: 10.1107/S2053229619003115 (PMC6452776; doi:10.1107/S2053229619003115)
Supplement: Supplementary file 4 [file c-75-00433-sup4.pdf]

STRUCTURAL CHEMISTRY

Volume 75 (2019)

Supporting information for article:

Polymorphism of the dinuclear Co^{III}–Schiff base complex [Co2(*o*-van-en)3]·4CH3CN (*o*-van-en is a salen-type ligand)

Anna Vráblová, Juraj Černák, Larry R. Falvello and Milagros Tomás

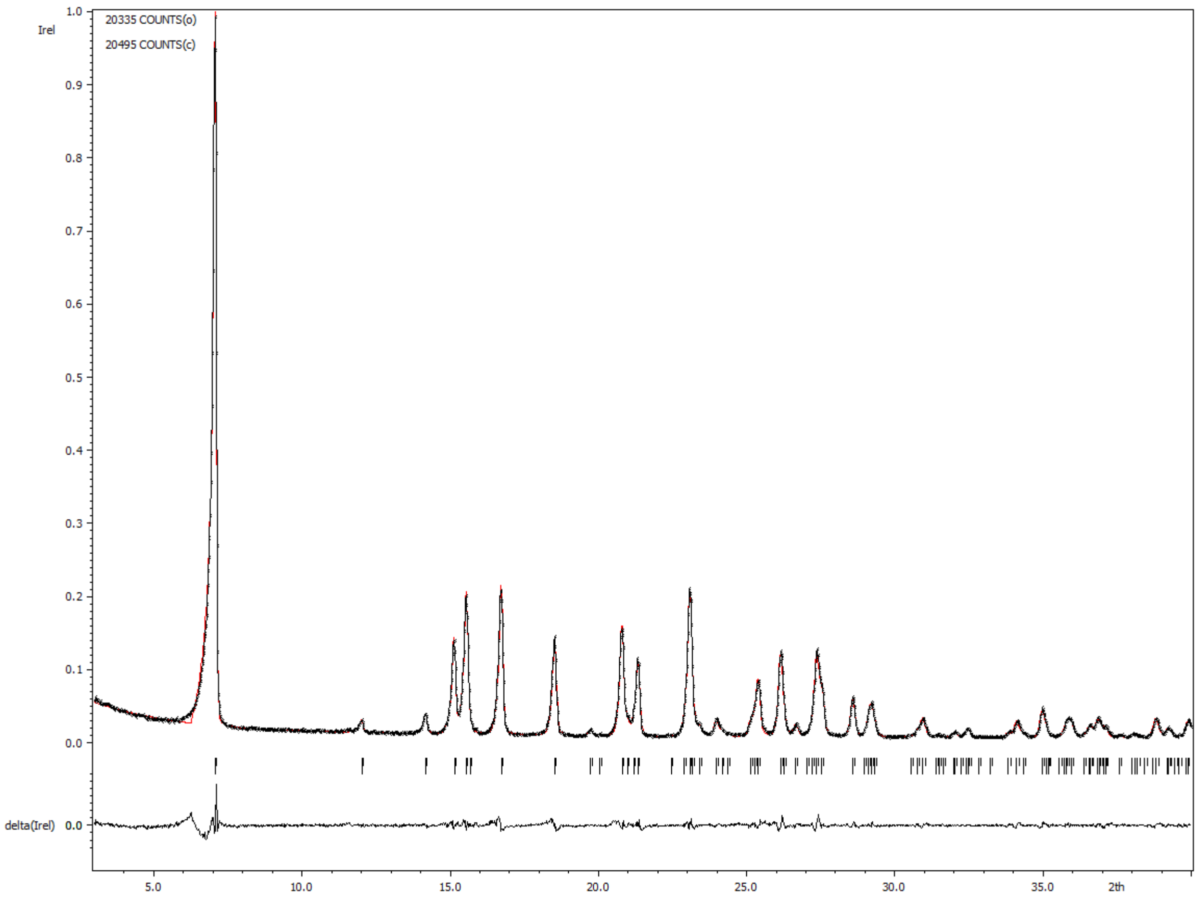

Figure S1. Results of Le Bail refinement of the powder diffraction pattern of **1**, with the starting unit-cell parameters taken from the known crystal structure of [Co(*o-van-en*)(H_2O)] (Jiang *et al.*, 2007). The refined cell parameters for orthorhombic space group *Pnma* are (the values reported by Jiang *et al.*, 2007 are given in the brackets) *a* = 8.9941(18) [8.9827 (6)] Å, *b* = 24.970(4) [24.8632 (16)] Å, *c* = 7.7011(9) [7.5784 (5)] Å. *R*(*p*) = 0.0541, *R*(*wp*) = 0.0754, goodness of fit = 1.91.

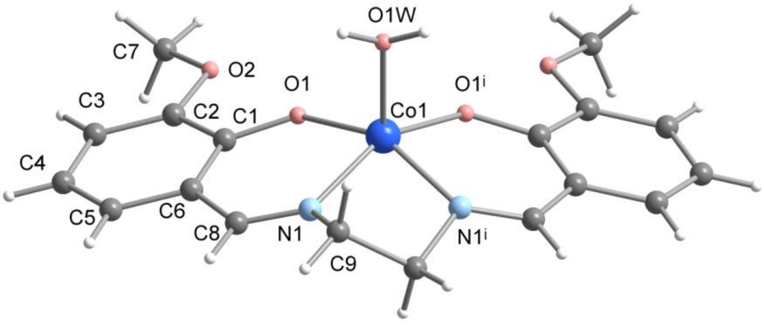

Figure S2. Molecular structure of **1** along with atom numbering scheme (Jiang *et al*., 2007). [Symmetry code: (i): *x*, 0.5 – *y*, *z*]

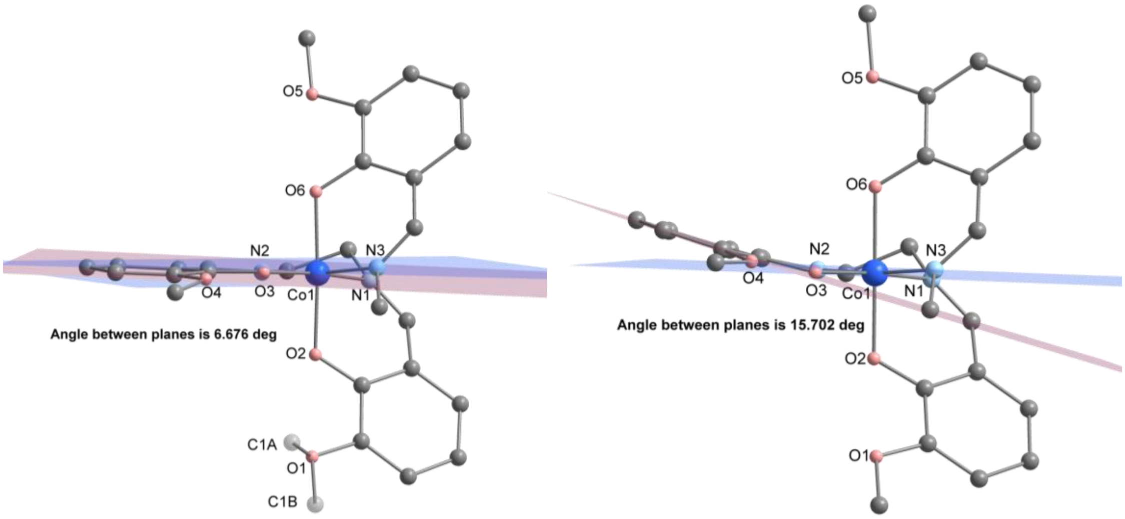

Figure S3. View of the angle between the phenyl ring of the ligand formed by C12 to C17 atoms (red) and the Co1-O3-N3-N1-N2 equatorial plane (blue) in compounds **2** (left) and **3** (right). We note that the methyl group of one methoxy group in **2** is positionally disordered (atoms C1A and C1B).

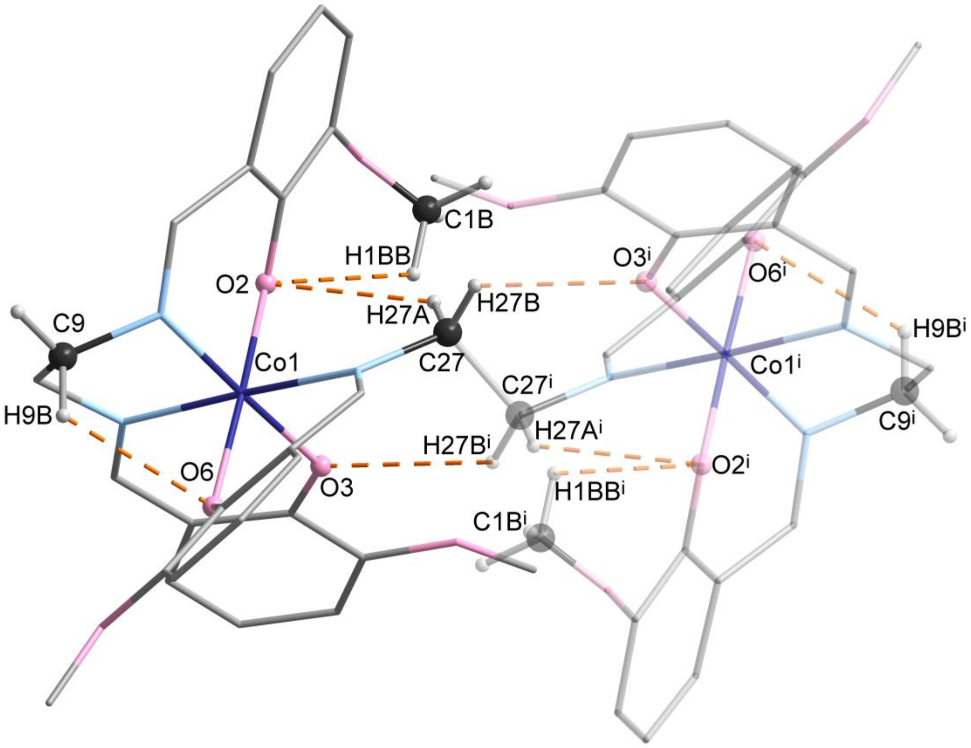

Figure S4. System of intramolecular close contacts (orange dashed lines) in **2**; disordered C1 atom is shown in its more populated position (C1B).

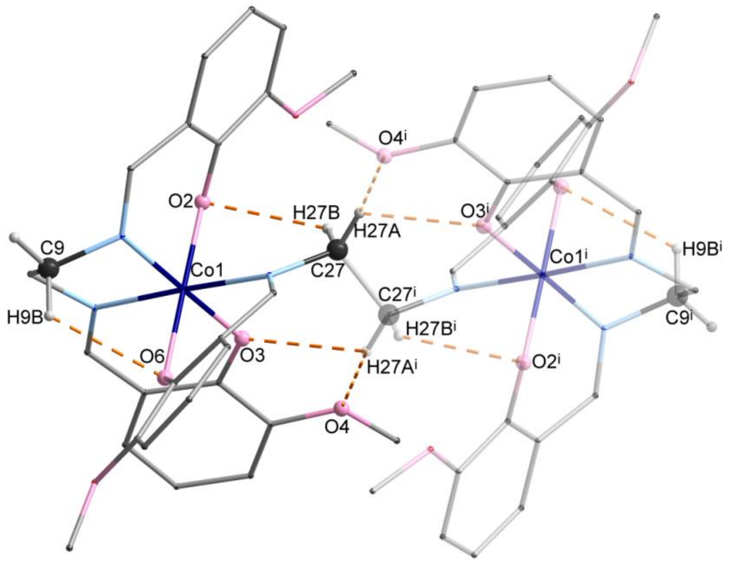

Figure S5. System of intramolecular close contacts (orange dashed lines) in **3**.

Table S1. Intermolecular interaction and close contacts of the π systems in **2**.

C-H···π	Distance C-H [Å]	Distance H··· Cg [Å]	Distance γ [°]	Angle C-H··· Cg [°]
$C21^{iii}$-$H21^{iii}$···Cg1	0.96(2)	2.91(3)	12.41	148(2)

Symmetry code: iii: *x*, 1/2 - *y*, 1/2 + *z*. Cg1 = center of gravity of the aromatic ring: C2-C3-C4-C5-C6-C7.

Table S2. Intermolecular interaction and close contacts of the π systems in **3**.

C-H···π	Distance C-H [Å]	Distance H··· Cg [Å]	Distance γ [°]	Angle C-H··· Cg [°]
C21-H21···$Cg2^{v}$	0.95	2.63	8.86	151

Symmetry code: v: 1 + *x*, *y*, *z*. Cg2 = center of gravity of the aromatic ring: C12-C13-C14-C15-C16-C17.

Hirshfeld surface analysis

As both **2** and **3** occur as disordered crystal structures Hirshfeld surfaces of dinuclear complex molecules were pictured for both disordered positions labelled A and B and compared separately (Fig. 7, Fig S6, Fig. 8, Fig. S7). In **2** the C1 methoxy group is positionally disordered over two positions with half occupancies. The reason for this disorder is obvious looking on the Fig. S6 displaying the Hirshfeld surface; the C1 methyl group in position A (atom C1A) forms a close contact to its symmetry (-1) generated congener (C1A···$C1A^{v}$ is 2.364(11) Å, v is –*x*, 1 - *y*, 1 - *z*) which manifests itself as a strong red spot. The situation for the second disordered position, labelled B is shown on the Figure 7; the above mentioned red spot is missing; on the other hand, the second disordered position of this methoxy group (O1-C1B) is close to MeCN solvate molecule which manifests itself by weak hydrogen bonding

interaction of the C-H···N type with N4 atom of the MeCN molecule (Tab. 3). We note that in this disordered position B an additional intramolecular hydrogen bonding interaction of the C-H···O type (C1B-H1BB···O2) is formed, too (Tab. 3). The above mentioned close contacts can be interpreted also by Fingerprint plots (Spackmann and McKinnon, 2002) generated by Hirshfeld surface of dinuclear complex molecule of **2** (disorder A and B) (Fig. S8). The closest contact observed in disorder A associated with C1A methoxy group results into short H···H, C···C and C···H contacts with short d_i+d_e sums up to 2.4 Å, displaying as two lobes at down-left part of the diagram. This feature is naturally missing in Fingerprint plot for disordered position B. On the other hand, in the case of second disordered position of **2** labelled B two already above mentioned additional weak hydrogen bonds of C-H···N and C-H···O types formed (Tab. 3) which leads to a certain increase of the O···H/H···O and N···H/N···H type close contacts displayed on corresponding Fingerprint plots (11.5 vs. 12.6 % and 9.3 vs. 9.5 %, respectively). The remaining red spots on the Hirshfeld surface for **2** correspond to close contacts and weak hydrogen bonding interactions of the C-H···N and C-H···O types displayed in Figure 7 and S6 as dashed lines; potential weak hydrogen bonds are gathered in Table 3. In addition, Figure 7 and S6 shows that the MeCN solvate molecules are located in the hollows on the surface of the complex molecules and thus contribute to better filling of the intermolecular space (packing) in the unit cell which in turn upon Kitajgorodskij contributes to the stabilization of the structure (Kitajgorodskij, 1965). We note that the calculations using PLATON indicated lack of any accessible voids in the crystal structure of **2**.

The calculated Hirschfeld surface for dinuclear complex molecule of **3** is shown on Fig. 8 and Fig. S7. In **3** the MeCN molecule involving N5 atom (as well as C31 atom) is positionally disordered with site occupation factors close to half (0.43 and 0.57 for N5A and N5B atoms, respectively); the left figure shows the situation with the MeCN molecule in A position (atoms N5A and C31A) and the right figure displays the same MeCN molecule in disordered positions B (atoms N5B and C31B). As the disorder appertain only the solvate molecule and, in addition, the two disordered positions are close to each other (N5A···N5B distance is 1.29 (3) Å), the Hirschfeld surfaces of the complex molecules for both situations are not affected. The only difference is that in the disordered position N5A a weak hydrogen bonding interaction C18-H18A···N5A is formed while a slightly stronger interaction C5-H5···N5B is formed in disordered position N5B (see red dashed lines on Fig. 8, Fig. S7). We note that the second interaction (N5B) is involved in the Table 4 while the first one involving N5A atom is not as the H18A···N5A contact (2.66 Å) is over the Platon limit for hydrogen bonding interaction. In line with this observation the position N5B is slightly more populated as the first one with N5A atom. The calculated fingerprint plots for **3** (Fig. S8) also reflect the differences for the two disordered positions of the MeCN molecules, i.e. concern mainly the N···H contacts as these are affected by the present disorder in the structure. These contacts are shorter in the fingerprint plots for N5B position with respects to N5A position.

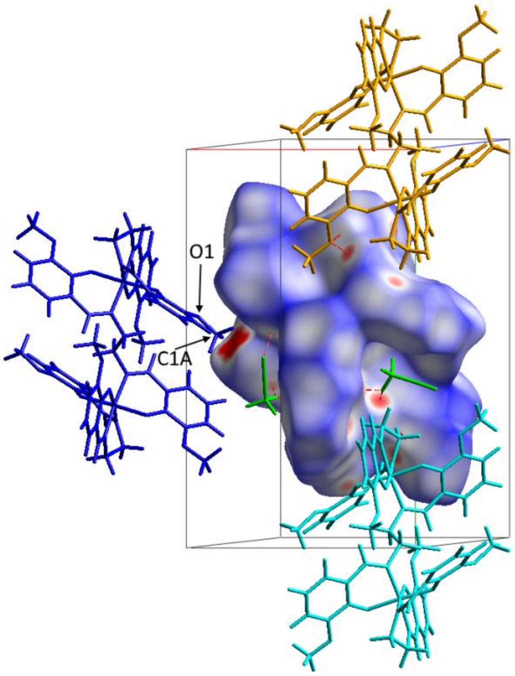

Figure S6. Hirshfeld surface diagram of the dinuclear complex molecule of **2** plotted over d_{norm} (normalised contact distance) from -0.4000 to 1.5000 a.u. Acetonitrile (MeCN) solvate molecules are displayed in green. Close contacts (O···H ≤ 2.60 Å, N···H ≤ 2.63 Å) are shown as red dashed lines: complex molecule with disordered C1A methyl group in position A. The neighbouring molecules are drawn using wire model in different colours in order to have a better insight on the contacts.

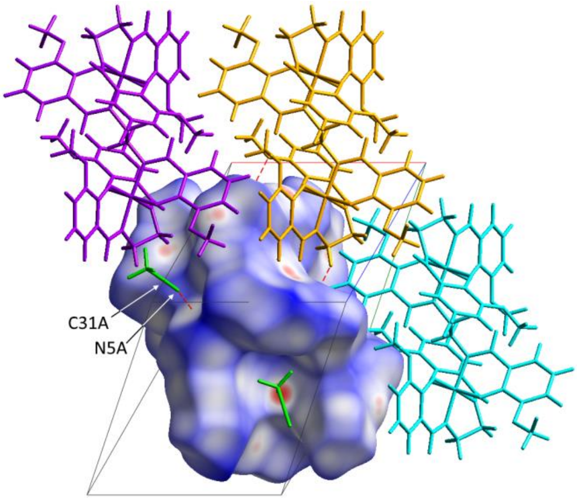

Figure S7. Hirshfeld surface diagram of the dinuclear complex molecule of **3** plotted over d_{norm} (normalised contact distance) from -0.4000 to 1.5000 a.u., disordered MeCN molecule in position A. MeCN solvate molecules displayed in green. Neighbouring complex molecules are shown in different colours using a wire model. Close contacts displayed in red dashed lines: O···H ≤ 2.60 Å, N···H ≤ 2.63 Å.

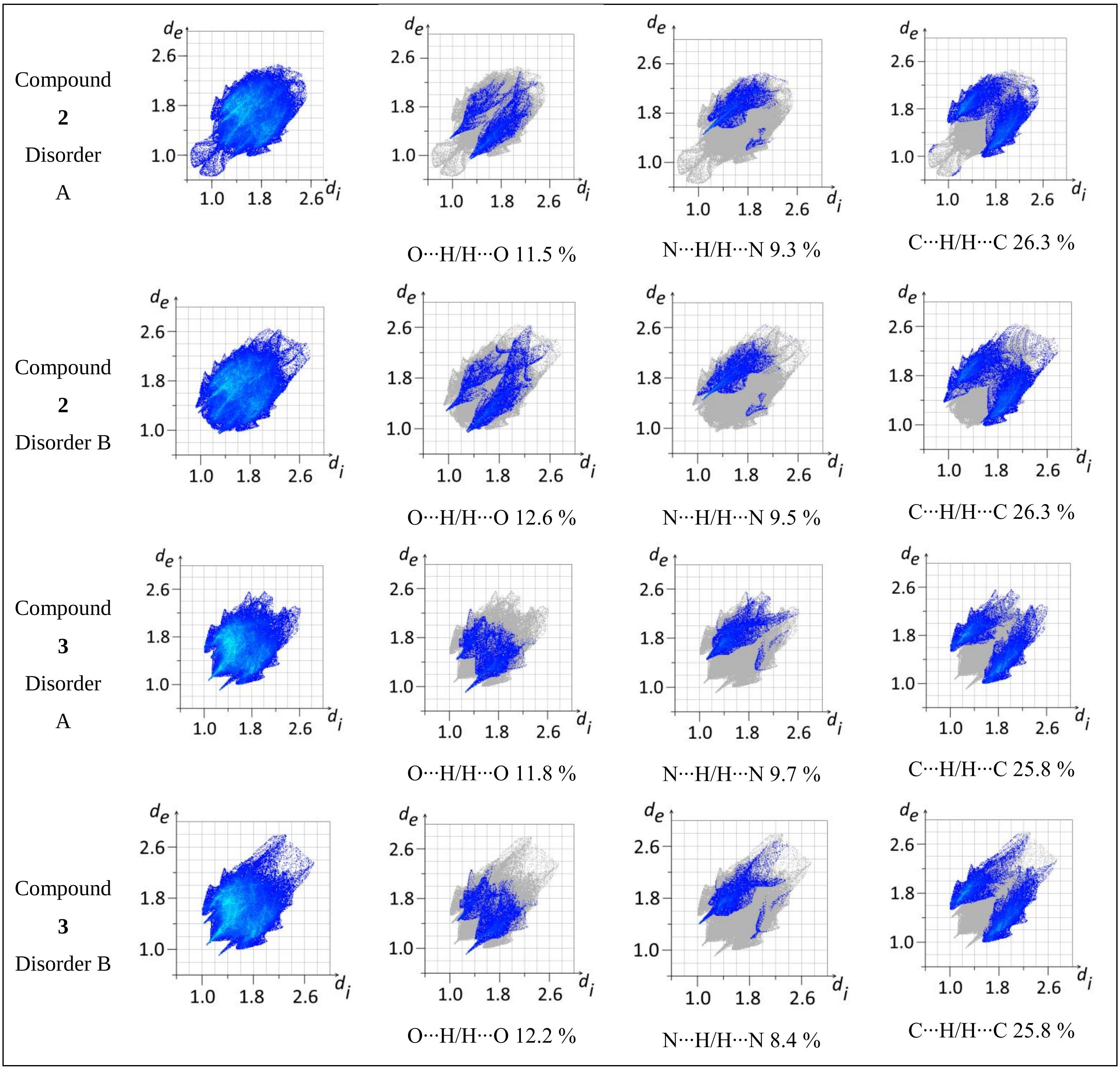

Figure S8. Fingerprint plots of dinuclear complex molecules of **2** and **3**, two different disorders for both structures. Contribution of the O···H/H···O, N···H/H···N and C···H/H···C short contacts is displayed for each situation.
